# Supplementary material for: Causal Relationship between Aging and Anorexia Nervosa: A White-Matter-Microstructure-Mediated Mendelian Randomization Analysis
Source: Biomedicines. 2024 Aug 16;12(8):1874. doi: 10.3390/biomedicines12081874 (PMC11351342; doi:10.3390/biomedicines12081874)
Supplement: Supplementary file 1 [file biomedicines-12-01874-s001.zip › Supplementary Figure/Figure S8/Leaveoneout plot of MR analysis for TL to ubm-b-1834.pdf]

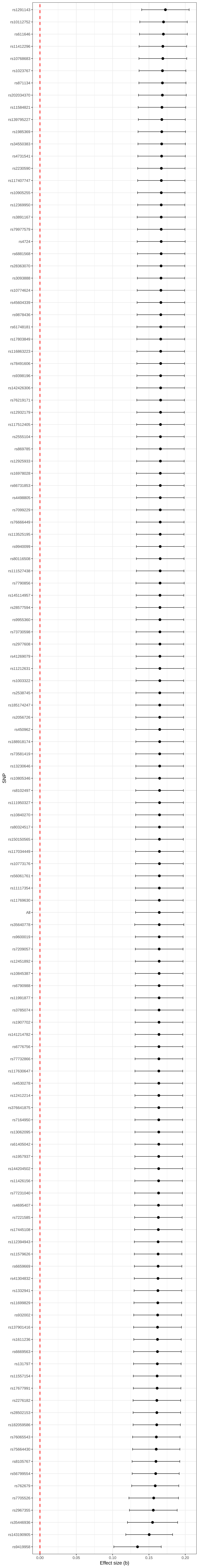

SNP
Effect size (b)
0.00
0.05
0.10
0.15
0.20
rs1291143
rs10112752
rs611646
rs11412296
rs10768683
rs1023767
rs871134
rs202034370
rs11584821
rs139795227
rs1985369
rs34550383
rs4731541
rs2230590
rs117407747
rs10905255
rs12369950
rs3891167
rs79977579
rs4724
rs6881568
rs28363070
rs3093888
rs10774624
rs45604339
rs9878436
rs61748181
rs17803849
rs116863223
rs78491606
rs9398196
rs142426306
rs76219171
rs12932179
rs117512405
rs2555104
rs869785
rs12925933
rs16978028
rs66731853
rs4498805
rs7099229
rs76666449
rs113525195
rs9940099
rs80116508
rs111527438
rs7790856
rs145114957
rs28577594
rs9955360
rs73730598
rs2977608
rs41269079
rs11212631
rs1003322
rs2538745
rs185174247
rs2056726
rs450962
rs188918174
rs73581419
rs13230646
rs10805346
rs8102497
rs111950327
rs10840270
rs80324517
rs150150565
rs117034449
rs10773176
rs56061761
rs11117354
rs11769630
All
rs35640778
rs9600019
rs7209057
rs12451892
rs10845387
rs6790988
rs11991877
rs3785074
rs1907702
rs141214782
rs6776756
rs77732866
rs117630647
rs4530278
rs12412214
rs376641875
rs7164950
rs13062095
rs61405042
rs1957937
rs144204502
rs11426156
rs77231040
rs4695407
rs7221585
rs17445108
rs112394943
rs11579626
rs6659669
rs41304832
rs1332941
rs11698829
rs932002
rs137901416
rs1611236
rs6669563
rs131797
rs11557154
rs17677991
rs2276182
rs28502153
rs182059586
rs76065543
rs75664430
rs8105767
rs56799554
rs762679
rs7705526
rs2967355
rs35446936
rs143190905
rs9419958
